# Validation of adipose lipid content as a body condition index for polar bears

**DOI:** 10.1002/ece3.956

**Published:** 2014-01-23

**Authors:** Melissa A McKinney, Todd Atwood, Rune Dietz, Christian Sonne, Sara J Iverson, Elizabeth Peacock

**Affiliations:** 1 Department of Biology, Dalhousie UniversityHalifax, Nova Scotia, B3H 4R2, Canada; 2 Great Lakes Institute for Environmental Research, University of WindsorWindsor, Ontario, N9B 3P4, Canada; 3 US Geological Survey, Alaska Science CenterAnchorage, Alaska, 99508; 4 Department of Biological Sciences, Arctic Research Centre, Aarhus UniversityRoskilde, DK-4000, Denmark

**Keywords:** Adipose tissue, biological validation, condition index, marine mammal, monitoring, quality control

## Abstract

Body condition is a key indicator of individual and population health. Yet, there is little consensus as to the most appropriate condition index (CI), and most of the currently used CIs have not been thoroughly validated and are logistically challenging. Adipose samples from large datasets of capture biopsied, remote biopsied, and harvested polar bears were used to validate adipose lipid content as a CI via tests of accuracy, precision, sensitivity, biopsy depth, and storage conditions and comparisons to established CIs, to measures of health and to demographic and ecological parameters. The lipid content analyses of even very small biopsy samples were highly accurate and precise, but results were influenced by tissue depth at which the sample was taken. Lipid content of capture biopsies and samples from harvested adult females was correlated with established CIs and/or conformed to expected biological variation and ecological changes. However, lipid content of remote biopsies was lower than capture biopsies and harvested samples, possibly due to lipid loss during dart retrieval. Lipid content CI is a biologically relevant, relatively inexpensive and rapidly assessed CI and can be determined routinely for individuals and populations in order to infer large-scale spatial and long-term temporal trends. As it is possible to collect samples during routine harvesting or remotely using biopsy darts, monitoring and assessment of body condition can be accomplished without capture and handling procedures or noninvasively, which are methods that are preferred by local communities. However, further work is needed to apply the method to remote biopsies.

## Introduction

Body condition is an important indicator of individual and population fitness, reproductive success, and overall health (Atkinson and Ramsay 1995; Robbins et al. 2012a). It is a measure of individual energy reserves, often expressed as percent body fat (Cattet et al. 2002; Stevenson and Woods 2006). Various nonlethal body condition indices (CIs) have been used in wildlife research, based on morphometric, biochemical, and physiological parameters. However, little consensus exists as to the most appropriate CI (Peig and Green 2010). Indeed, many CIs have not been thoroughly tested for accuracy, precision, sensitivity, biological significance, or the range of circumstances under which they may be valid (Cook et al. 2001).

In large mammals, body condition has mainly been reported using morphometric-based CIs including body mass, length, skull size, girth, and combined measures including body mass index (BMI), body condition index (BCI), and storage energy (Cattet et al. 2002; Stevenson and Woods 2006; Molnár et al. 2009). Subjective fatness indices (FI) from physical and/or visual examination have also been used (Stirling et al. 1989). Some of these CIs indicate biological significance, supported by correlative associations with measures of overall health, such as litter mass (Rode et al. 2010). Direct body composition estimates from isotope dilution or bioelectrical impedance analysis (BIA) have also been validated against total body fat and measures of biological significance in various species (Farley and Robbins 1994; Jacobs et al. 2012; Robbins et al. 2012a). However, some of these CIs exhibit high variability and/or subjectivity, preventing objective assessment of precise large-scale spatial or long-term temporal trends in body condition of free-ranging wildlife.

Although many analyses (e.g., tooth-based aging; Calvert and Ramsay 1998) still require animal handling, a more suitable CI would be minimally or noninvasive (e.g., remote biopsy darting during genetic sampling for mark-recapture population estimates) or from regularly harvested populations (e.g., subsistence hunting), compare favorably with other CIs, show biological significance, and be relatively inexpensive and simple so that it could be determined routinely from sufficient numbers of individuals and populations to infer trends. An ideal approach would be an *in vitro* biochemical metric that could be measured on tissues already being collected by wildlife biologists or during regular harvesting, including hair, skin, adipose and/or blood. Specifically, adipose lipid content has been proposed based on studies linking it with total fatness in several mammalian species (e.g., Shier and Schemmel 1975; Beck et al. 1993; Gomez-Campos et al. 2011) and preliminary studies in polar bears *Ursus maritimus* (Thiemann et al. 2006; Stirling et al. 2008). These studies have demonstrated lipid content variation with FI and, as expected, with season. Lipid content analysis is inexpensive, precise, and straightforward and thus applicable on a broad scale. However, robustness of the laboratory method and validation of lipid content relative to multiple accepted CIs and other indicators of health have not been fully evaluated.

Polar bears from the Southern Beaufort Sea (SB) and elsewhere have exhibited body condition declines associated with rapid climate warming, decreasing sea ice habitats, and reduced access to their main prey, seals (Stirling et al. 1999; Obbard et al. 2006; Rode et al. 2010, 2012). These condition changes preceded changes in subpopulation health, including reduced litter mass, litter size, survival, and overall subpopulation size (Derocher and Stirling 1994, 1996; Stirling et al. 1999; Regehr et al. 2007; Rode et al. 2010). Extensive datasets from well-studied subpopulations are useful to calibrate potential health indices for use in other subpopulations with poor research access (Vongraven et al. 2012). Here, we examine a substantive SB polar bear adipose capture biopsy archive, for which detailed biological and capture information has been recorded, including established CIs and health parameters. This dataset provides an opportunity to thoroughly evaluate adipose lipid content as a CI. In response to some community, management, and scientist concerns (with regard to, e.g., capture, handling, collaring, chemical immobilization,), noninvasive sampling methods have been proposed (Peacock et al. 2011; Vongraven et al. 2012) and developed for polar bears and other wildlife (e.g., Fedigan 2010; Herreman and Peacock 2013; Pagano et al. 2014). Here, we also test the applicability of lipid content to recently collected SB polar bear remote biopsies for which no established CIs besides FI could be obtained. Application of the lipid content CI to remotely taken biopsies would minimize sampling invasiveness, increase sample sizes and potentially increase the geographic scope of sampling in more remote areas with poor research access. More generally, a validated lipid content CI could be used to expand large-scale (circumpolar) and long-term (retrospective) assessment capabilities.

Development of a calibrated index of polar bear health based on a rapid and reliable assessment method will help satisfy the need for increased monitoring (Vongraven et al. 2012) due to rapidly changing ice conditions for polar bears (Overland and Wang 2013), and would be applicable to other wildlife, which store lipid in subcutaneous adipose/blubber depots such as marine mammals. Here, we assess the validity of adipose lipid content as a quantitative CI, building on previous preliminary work (Thiemann et al. 2006; Stirling et al. 2008). We focus on remote biopsy, capture biopsy, and harvest samples to assess the lipid content CI in terms of accuracy, precision, sensitivity, influence of biopsy depth, and storage condition, and compare lipid content to established CIs and demographic and ecological parameters.

## Materials and Methods

### Sample collection

We collected subcutaneous adipose tissue samples with a 6 mm biopsy punch from the rump region of 550 anesthetized polar bears captured between 141–157°W on the sea ice off of northern Alaska (i.e., the southern Beaufort Sea) between 2004–2011. We sampled bears in the spring (*n *=* *474) from March to mid-May. In 2008–2010, we sampled additional SB bears in the late summer–early fall (herein referred to as “fall”) (*n *=* *76) from August to October. We recorded position (lat/long) of capture, sex, girth, total length, FI, mass, BMI, BIA (resistance, ohms), skull width and length, number and age class of accompanying cubs, cub sex, weight, skull width and/or length (Rode et al. 2010). We determined ages for first-time captured bears by counting the growth layer groups in the cementum of a vestigial premolar tooth (Calvert and Ramsay 1998) and classified bears by age/sex class: cubs-of-the-year (C0), yearlings (C1), 2-year-old dependent cubs (C2), subadult (independent 2-, 3- and 4-year-old) females and males (SF, SM) and adult (5-year and older) females and males (AF, AM) (Stirling et al. 1975).

We obtained adipose biopsies from 96 SB bears sampled by remote biopsy dart in fall 2011 (*n *=* *45) and spring (*n *=* *24) and fall 2012 (*n *=* *27) (Pagano et al. 2014). As these bears were not tranquilized and handled, we only recorded position, sex, number, and age class of accompanying cubs, estimated age class and remotely ascertained FI. In fall 2011 and spring 2012, we collected samples from various body regions, but largely near the shoulder area. To obtain biopsies more comparable to those from captured bears, fall 2012 darting targeted the rump region.

For long-term storage, we kept all SB biopsies frozen (−80°C). We sent skin and hair, removed prior to lipid analysis, to Wildlife Genetics International Inc. (Nelson, BC, Canada) to genetically identify all captured and remotely darted bears (Pagano et al. 2014). We used additional adipose samples acquired for other projects from polar bears harvested in the Baffin Bay (BB) and East Greenland (EG) subpopulations in the laboratory validation portion of this study. For these harvested tissues, which were stored at −20°C, we subsampled from the innermost tissue section for lipid analysis.

### Adipose lipid content analysis

We extracted lipid from biopsies and harvest samples and reported it as the ratio of extracted lipid relative to initial wet weight of adipose tissue, in percentage. Briefly, biopsies were measured by ruler to the nearest millimeter, transferred into tared glass vials, and weighed to the nearest *μ*g (Mettler-Toledo XP26 microbalance with ergo clip adaptor; Columbus, OH, USA). Chloroform was added containing 0.01% butylated hydroxytoluene (BHT) as an antioxidant. Samples were flushed with nitrogen, capped, and stored at −20°C until lipid extraction. Lipid was extracted as described previously (Budge et al. 2006) with modifications. Samples were quantitatively transferred to a glass tube, spiked with 5-*α*-cholestane as internal standard (100 *μ*L of 20 mg/mL), and homogenized by Teflon pestle attached to an electric stirrer. Lipids were extracted twice using 8:4:3 chloroform/methanol/water containing BHT, at a volume/weight ratio of 20:1 solvent/sample. Isolated lipids were completely evaporated of solvent, then weighed by microbalance. Extracted fatty acids (FAs) were derivatized to FA methyl esters (FAMEs) and analyzed by gas chromatography with flame ionization detection (GC-FID) to quantify internal standard recoveries.

### Laboratory method validation

We investigated accuracy and precision using the National Institute of Standards and Technology (NIST) standard reference material SRM 1945 (whale blubber homogenate) for which a consensus value of percent lipid (“total extractable organics”) from interlaboratory comparison exercises has been published (Kucklick et al. 2010). A ˜0.05 g subsample of SRM 1945 (similar to capture and remote biopsy weights of ˜0.01–0.3 g) was analyzed with each sample batch or 64 times in total. We tested lipid recoveries for individual samples using the internal standard. We examined the influence of biopsy depth by analyzing equal length inner (near muscle) and outer (near skin) sections of a subset (*n *=* *21) of the spring 2010 capture biopsies, as both capture and remote biopsies were generally not taken at full depth. With a larger adipose sample from a harvested BB polar bear and with SRM1945, we examined the sensitivity of the extraction to sample size, that is, whether the small sizes of the capture and remote biopsies were sufficient for gravimetric lipid analysis.

We measured lipid content from a 1984–2011 EG dataset and a 2004–2011 SB capture biopsy dataset. As we only recently (2011–2012) analyzed both datasets, interannual variation in lipid content may suggest changes in CI over time or potential impacts of long-term storage on lipid content. For example, storage effects can only result in lower lipid content in older samples and not the reverse pattern. However, CI effects can lead to variation in either direction. Thus, if we identify lower lipid content in the newer samples, the main driver must be CI changes. If we identify higher lipid content in newer samples or no temporal trend in either direction, then storage and/or CI effects could be occurring.

### Calculation of established CIs and relationships to adipose lipid content

We calculated BMI (body mass (kg)/(length (m))^2^), BCI (standard residuals from regression of body mass (kg) on length (m), ranging from −3.0 to +3.0), and storage energy (*x*_1_(body mass (kg)) – *x*_2_(length (m))^3^, where the coefficients *x*_1_ and *x*_2_ are sex/age class-specific) from published methods (Cattet et al. 2002; Stevenson and Woods 2006; Molnár et al. 2009). Some negative storage energy values that occurred, likely because the coefficients were generated for a different polar bear subpopulation (i.e., Western Hudson Bay; WH) (Molnár et al. 2009), were discarded. Although FI values (from 1 to 5, where 1 is leanest and 5 most obese) were sometimes reported as half-integers (i.e. 1.5, 2.5, 3.5, 4.5), we down-assigned these values to whole integers (1, 2, 3, 4, respectively) for consistency with previous studies (Stirling et al. 2008). We also included skull width as a CI since skulls may have a variable fat layer reflecting body condition, and as previous studies showed skull width declines over time (Rode et al. 2010, 2012).

We performed nonparametric tests for lipid content and other CIs, since measures frequently did not meet the assumption of homogeneity of variances even after data transformation. We tested inner and outer subsections for differences by Wilcoxon matched pairs test. We tested sample size and storage time (collection year) influences on lipid content using Spearman's correlation analysis. We considered the potentially confounding variables of sex/age class. First, we tested lipid content for sex/age class differences, then grouped accordingly, and compared CIs. We compared FI to other CIs using Kruskal–Wallis ANOVA and post-hoc Mann–Whitney *U*-tests. We compared all other CIs using Spearman's correlation analysis. We used simple linear regression with AFs and SFs to determine whether BIA (resistance) was correlated with transformed lipid content and conducted an ancillary analysis using Kruskal–Wallis ANOVA to determine whether gut fill (i.e., full, partial, empty) from recent meals inflated BIA values (Hilderbrand et al. 2000). We analyzed gut fill using samples from 2012, the only year with gut fill data. We reported all data as mean ± SE.

### Ecological patterns in relation to lipid content

We assessed whether lipid content varied with biological and ecological parameters, including sex/age class, reproductive status, season, and year (Thiemann et al. 2006; Stirling et al. 2008). We tested sex/age class and seasonal differences using Kruskal–Wallis ANOVA and post-hoc Mann–Whitney *U*-tests. We also assessed more fine-scale seasonal variation by examining mean lipid content variation with ordinal date of sampling. We examined temporal trends for SB and EG using correlation analysis, and for SB, we assessed interannual lipid content variation between 2004–2011 using Kruskal–Wallis ANOVA and Mann–Whitney *U*-tests.

We used generalized linear models to investigate the relationship between lipid content and sea ice availability for SB AMs and AFs. We used two measures of sea ice availability: duration of the summer melt season (*Melt*) for the entire SB and the finer-scale measure of ice-free days (*IFD*) over the continental shelf (Appendix S1). We also included year, age (*Age*), ordinal date (*Odate*) of sampling, and number of dependent cubs (*Cub*; for AFs) as continuous covariates to control for other variables that may influence polar bear body condition (e.g., Rode et al. 2012). We developed biologically plausible candidate models and used Akaike's Information Criterion for small sample sizes (AIC_c_) to determine top models for each sex class (Appendix S1). We did not include year and ice availability metrics in the same model because they may reflect different temporal scales (Rode et al. 2012).

## Results

### Lipid content of capture biopsies

Adipose lipid content of the 2004–2011 SB capture biopsies averaged 43.2 ± 0.7% (Table 1) and differed among age/sex classes (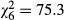, *P* < 0.001). AMs had the lowest lipid content (36.2 ± 1.1%, *P* < 0.001). AFs had lower lipid content (44.4 ± 1.2%) than C0s and SFs (*P* ≤ 0.003), and lipid content did not significantly differ among C0s, C1s, C2s, SFs, and SMs (50.7 ± 1.2%). Therefore, lipid content results are subsequently reported separately for AFs, AMs, and immature bears (comprising C0s, C1s, C2s, SFs, and SMs).

**Table 1:** Adipose lipid content ± SE of capture and remote biopsies from polar bears from the Southern Beaufort (SB) Sea subpopulation sampled from 2004–2011.

	Adipose lipid content (%)			
	All bears	Adult females	Adult males	Immature bears
Capture biopsies				
All years (2004–2011)	43.2 ± 0.7 (*n *=* *550)	44.4 ± 1.2^a^ (*n *=* *199)	36.2 ± 1.1^b^ (*n *=* *197)	50.7 ± 1.2^c^ (*n *=* *154)
All years-spring (2004–2011)	41.0 ± 0.8 (*n *=* *474)	41.8 ± 1.3 (*n *=* *161)	36.0 ± 1.1 (*n *=* *191)	48.4 ± 1.5 (*n *=* *122)
All years-fall (2008–2010)	56.9 ± 1.3 (*n *=* *76)	56.0 ± 2.0 (*n *=* *38)	51.4 ± 9.7 (*n *=* *6)	58.3 ± 1.6 (*n *=* *32)
2004-spring	39.0 ± 1.9	39.1 ± 3.0	30.0 ± 2.9	45.9 ± 3.3
2005-spring	44.8 ± 2.0	45.5 ± 3.5	38.9 ± 2.6	56.3 ± 3.8
2006-spring	37.5 ± 2.2	45.3 ± 2.9	31.7 ± 3.3	40.6 ± 4.6
2007-spring	36.7 ± 1.8	36.2 ± 2.3	32.1 ± 2.2	53.5 ± 5.6
2008-spring	45.6 ± 1.9	46.0 ± 4.4	42.3 ± 2.7	50.7 ± 2.2
2009-spring	38.6 ± 2.0	39.5 ± 4.7	38.2 ± 2.6	38.8 ± 3.7
2010-spring	39.8 ± 2.5	40.3 ± 3.7	31.5 ± 3.4	50.1 ± 5.4
2011-spring	47.8 ± 2.2	47.2 ± 3.8	46.5 ± 4.4	48.9 ± 3.5
2008-fall	57.7 ± 1.4	57.2 ± 2.8	57.5 ± 0.0	58.0 ± 1.6
2009-fall	55.5 ± 2.8	54.4 ± 3.4	48.4 ± 16.0	59.4 ± 4.8
2010-fall	56.6 ± 3.9	56.6 ± 3.9		
Remote biopsies				
All years (2011–2012)	20.9 ± 1.3 (*n *=* *96)	22.8 ± 1.9^a^ (*n *=* *45)	18.0 ± 1.8^a^ (*n *=* *37)	22.4 ± 4.4^a^ (*n *=* *14)
All years-spring (2012)	26.1 ± 3.1 (*n *=* *24)	33.4 ± 4.7 (*n *=* *11)	19.6 ± 4.0 (*n *=* *9)	20.7 ± 6.6 (*n *=* *4)
All years-fall (2011–2012)	19.1 ± 1.4 (*n *=* *72)	19.3 ± 1.7 (*n *=* *34)	17.4 ± 2.0 (*n *=* *28)	23.2 ± 5.8 (*n *=* *10)
2011-fall	19.5 ± 1.8	19.8 ± 2.3	16.0 ± 2.6	30.4 ± 6.9
2012-fall	18.4 ± 2.2	18.4 ± 2.3	19.6 ± 3.1	15.9 ± 8.8

Different letters denote significant differences in lipid content among age/sex classes.

### Lipid content of remote biopsies

Lipid content of the 2011–2012 SB remote biopsies averaged 20.9 ± 1.3% (Table 1) and did not significantly differ among AMs, AFs, and immature (subadults only as cubs were not sampled) bears (*P* = 0.26). Since lipid content was lower for remote versus capture biopsies, and varied with biopsy depth (see *Validation of the laboratory method*), we checked whether biopsy lengths differed between the two biopsy types. Capture and remote biopsies were not significantly different in length (2.3 ± 0.1 cm and 2.1 ± 0.1 cm, respectively; *P* = 0.07). Fall 2012 remote biopsy collections targeted the rump region and averaged a higher length of 2.3 ± 0.3 cm, but still had lower lipid content (18.4 ± 2.2%) than capture biopsies.

### Validation of the laboratory method

**Figure 1:**
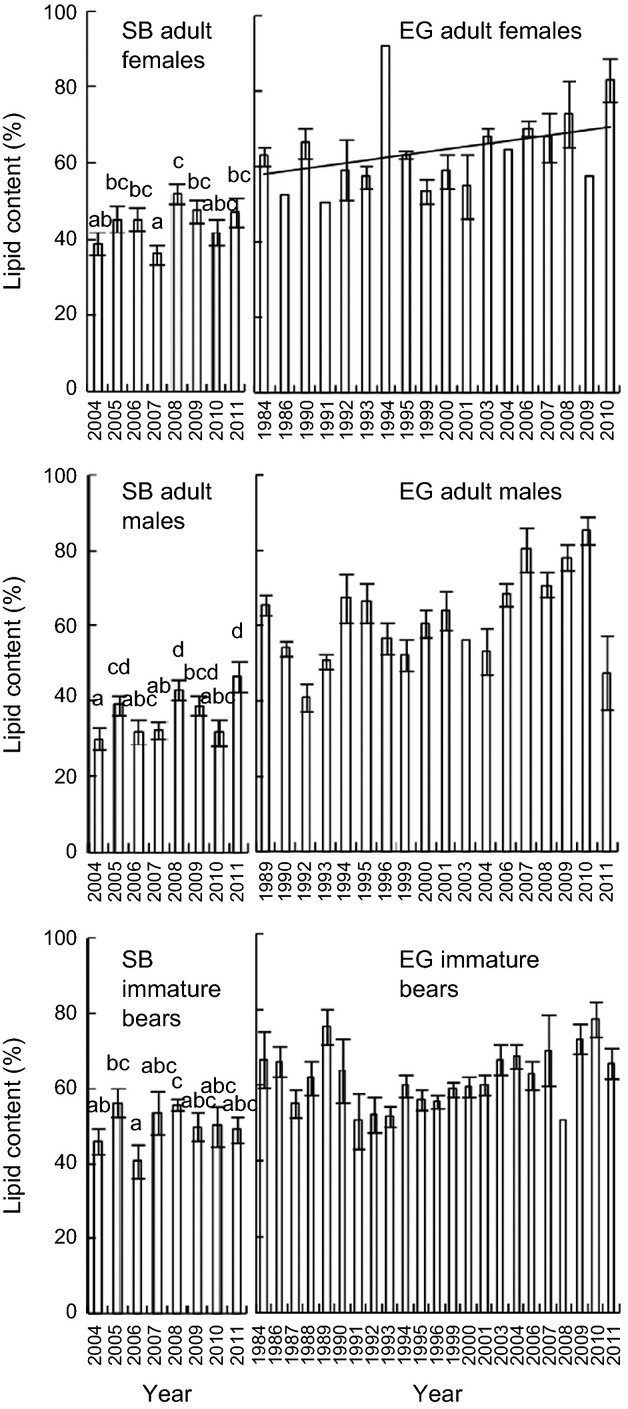
Adipose lipid content (±SE) of adult female, adult male and immature polar bears sampled in the Southern Beaufort Sea (SB; 2004–2011) and East Greenland (EG; 1984–2011) subpopulations. Different letters indicate significant differences in SB lipid content. Trendlines indicate significant linear trends.

The lipid extraction method was determined to have high accuracy and precision, with little sensitivity to sample size (Appendix S1, Figure S1). Capture biopsies were higher in lipid content for inner versus outer adipose tissue (*z *=* *4.0, *P* < 0.001; Figure S2). Mean SB adipose lipid content exhibited no significant trend from 2004–2011 (*P* > 0.26) for all age/sex classes, but did differ among years (*χ*^2^ > 14.1, *P* ≤ 0.05; Fig. 1). For harvested AF EG bears, lipid content significantly increased from 1984–2010 (*r*^2^* *=* *0.22, *P* = 0.05; Fig. 1). The pattern was similar but not significant for AMs (1989–2011) and immature (1984–2011) EG bears (*P* ≥ 0.09).

### Comparison with other metrics

**Figure 2:**
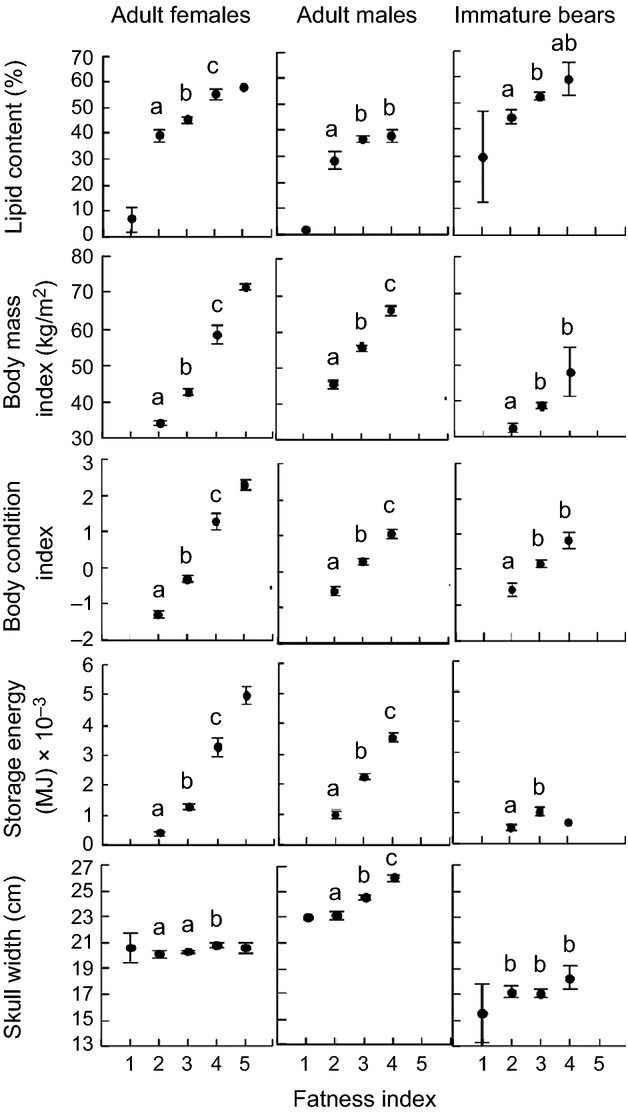
Adipose lipid content, body mass index, body condition index, storage energy, and skull width (±SE) by fatness index for polar bears from the Southern Beaufort Sea subpopulation sampled during capture in 2004–2011. Significant differences indicated by different letters. Missing data points or those without letters were not statistically compared due to ≤2 samples for each sex/age class.

For AF, AM, and immature bears, capture biopsy lipid content differed by FI scores (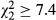, *P* ≤ 0.02; Fig. 2). Significantly higher lipid content was found in AFs assigned to FI 4 (55.3 ± 2.3%) versus 3 (45.4 ± 1.4%), and FI 3 versus 2 (39.3 ± 2.4%; *P* < 0.03). Lipid content did not differ for AMs assigned to FI 4 versus 3 (38.2 ± 2.3% and 37.0 ± 1.3%, respectively), but was higher for FI 3 than FI 2 (28.6 ± 3.4%; *P* = 0.007). Similarly, lipid content did not differ for immature bears of FI 4 versus 3 (59.4 ± 6.5% and 52.7 ± 1.5%, respectively), but was higher for FI 3 than FI 2 (44.8 ± 2.7%; *P* = 0.01). Although sample sizes were too small to statistically compare polar bears assigned to FI 1 or 5 (*n *≤* *2), the pattern was consistent, that is, lower lipid content with lower FI scores.

Differences in established CIs across FI scores were also significant for AFs, AMs, and immature polar bears (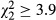, *P* ≤ 0.05; Fig. 2), except for skull width in immature bears. Like lipid content, BMI, BCI, storage energy, and skull width were higher in AFs assigned to FI 4 versus 3, and all but skull width were higher in AFs assigned to FI 3 versus 2 (*P* < 0.001). Again, although sample sizes were too low to statistically compare AFs assigned to FI 5, these bears were higher in BMI, BCI, and storage energy (but not skull width) than FI 4 bears. No BMI, BCI, or storage energy data were available for AFs assigned to FI 1. Skull width was not higher in AFs assigned to FI 2 versus 1. Similar to lipid content, the established CIs were higher in AMs, and in immature bears except for skull width, assigned to FI of 3 versus 2 (*P* ≤ 0.05). For AMs only, these CIs were also significantly higher for FIs of 4 versus 3 (*P* < 0.001).

**Figure 3:**
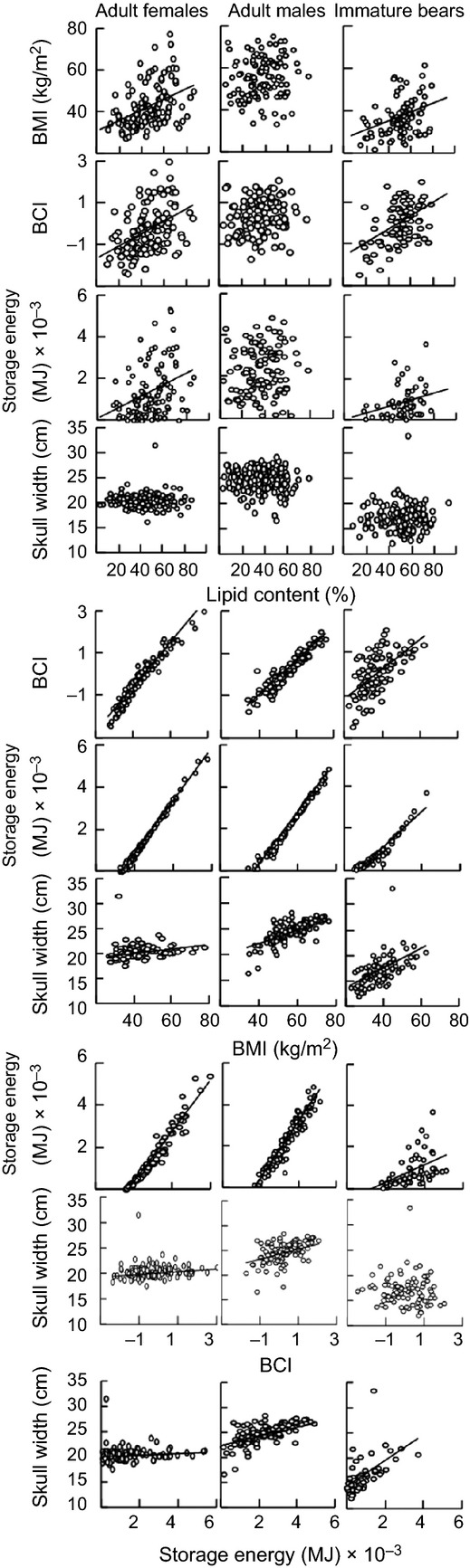
Relationships between adipose lipid content, body mass index (BMI), body condition index (BCI), storage energy, and skull width for polar bears from the Southern Beaufort Sea subpopulation sampled in 2004–2011. Trendlines denote significant regression lines.

Adipose lipid content of AFs was a significant predictor of BMI (Spearman *r*^2^* *=* *0.21, *P* < 0.001), BCI (*r*^2^* *=* *0.22, *P* < 0.001) and storage energy (*r*^2^* *=* *0.13, *P* < 0.001), but not skull width (Fig. 3). In immature bears, it was also a significant predictor of BMI (*r*^2^* *=* *0.13, *P* < 0.001), BCI (*r*^2^* *=* *0.20, *P* < 0.001) and storage energy (*r*^2^* *=* *0.11, *P* = 0.01), but not skull width. However, in AMs, lipid content was not significantly correlated with BMI, BCI, storage energy or skull width (*P* ≥ 0.07). Mean BIA values for AFs and SFs were not predicted by lipid content (*r*^2^ = −0.01, *P* = 0.67), and the extent of gut fill did not significantly inflate BIA values (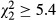, *P* = 0.07).

The BMI, BCI, and storage energy indices were significantly and strongly correlated with one another for AFs (*r*^2^ ≥ 0.95, *P* < 0.001) and AMs (*r*^2^ ≥ 0.90, *P* < 0.001). Storage energy and BMI were strongly correlated (*r*^2^* *=* *0.90, *P* < 0.001) in immature bears; however, the relationship was not as strong for storage energy and BCI (*r*^2^* *=* *0.38, *P* < 0.001) and for BCI and BMI (*r*^2^* *=* *0.38, *P* < 0.001). Skull width was significantly, but not strongly correlated with BMI, BCI, and storage energy for AFs (*r*^2^ ≥ 0.04, *P* < 0.04) and AMs (*r*^2^ ≥ 0.30, *P* < 0.001). Skull width was significantly correlated with BMI and storage energy (*r*^2^ ≥ 0.40, *P* < 0.001), but not BCI, in immature bears.

For remote biopsies, lipid content comparisons were assessed with remotely scored FI. However, due to limited sample sizes, only polar bears of FI 3 and 4 could be compared and only for AFs and AMs. Lipid content was not significantly different in AFs of FI 4 (24.0 ± 5.7%) versus 3 (22.5 ± 2.0%; *P* = 0.97), nor in AMs with FI 4 (21.3 ± 3.3%) versus 3 (14.5 ± 2.2%; *P* = 0.11).

### Ecological and demographic patterns

**Figure 4:**
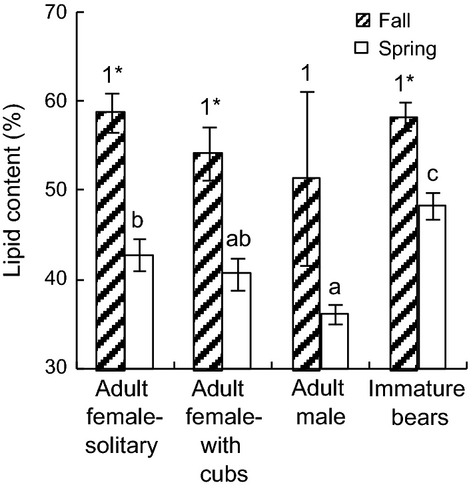
Adipose lipid content (±SE) by age/sex class of polar bears from the Southern Beaufort Sea subpopulation captured from 2004 to 2011. Different numbers indicate significant differences in fall lipid content among demographic groups, different letters indicate significant differences in spring lipid content among groups, and asterisks indicate higher lipid content in fall than spring bears within a group.

**Figure 5:**
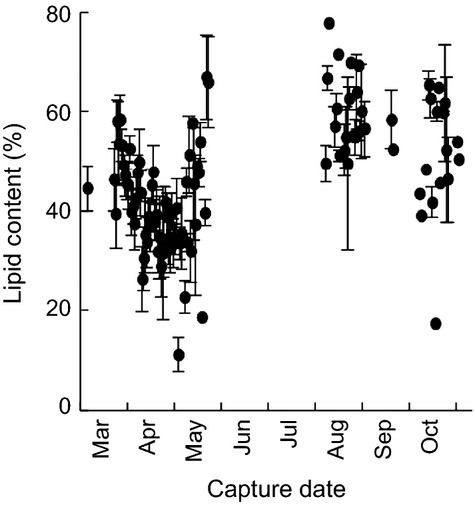
Adipose lipid content (±SE) by capture date for polar bears from the Southern Beaufort Sea subpopulation sampled from 2004 to 2011.

Although there was a pattern of decreasing lipid content in the fall from solitary AFs to AFs with cubs to AMs, there were no statistically significant differences (*P* = 0.51; Fig. 4). However, the sensitivity of the dataset to detect differences in lipid content in the fall may have been low as a result of low fall samples sizes relative to spring collections (Table 1). In the spring, lipid content was higher in immature bears than in AMs and AFs (*P* = 0.008) and in solitary AFs than in AMs (*P* = 0.002). Fall versus spring lipid content was significantly higher for solitary AFs (16% higher), AFs with cubs (13%) and immature bears (10%; *P* < 0.001). When examined by capture date, lipid content appeared to decline from March to May (˜55–35%), increase in May (to >60%), and decline slightly from September to November (Fig. 5).

As previously described, adipose lipid content of AF, AM, and immature SB polar bears exhibited no time trends from 2004–2011, whereas for AF EG polar bears, lipid content increased from 1984–2010. No time trends were found for skull width of SB AFs and AMs between 2004–2011 (*P* > 0.10). However, skull width declined by 0.20 cm yr^−1^ in immature bears.

For SB adults, spring lipid content was inversely correlated with duration of the reduced sea ice extent period during the previous open-water season (summer/fall). *Melt* was the primary driver of lipid content differences over time for AFs: five models were included in the top model set (Table 2), each retaining *Melt* as a variable with a normalized Akaike weight of 0.31. For AMs, four models comprised the top model set (Table 2); *IFD* and *Odate* were retained in all four models with normalized Akaike weights of 0.34 and 0.32, respectively. *Melt* was retained in two of the top models for AMs. Small sample sizes for immature bears precluded a similar analysis.

**Table 2:** Top models (Akaike's Information Criterion values [AIC_c_], Akaike weights [*w*_*i*_], and *P*-values) of variation in lipid content for adult polar bears in the southern Beaufort Sea subpopulation, 2004–2011, where *melt* is duration of summer melt season, *IFD* is ice-free days, *age* is bear age, *Odate* is ordinal sampling date, and *cub* is number of dependent cubs.

Model	ΔAICc	*w*_*i*_ (model)	*P* (model)
Adult females			
*Melt*	0.00	0.15	0.010
*Melt, IFD*	1.17	0.08	0.021
*Odate, Melt*	1.73	0.06	0.036
*Melt, Cub*	1.86	0.06	0.040
*Age, Melt*	1.89	0.06	0.131
Adult males			
*IFD, Melt, Odate*	0.00	0.29	<0.0001
*Odate, IFD*	0.69	0.20	<0.0001
*Age, Odate, IFD*	1.09	0.17	0.0002
*Age, Odate, Melt, IFD*	1.91	0.11	0.0003

## Discussion

We conclude that adipose lipid content is a potentially effective biochemical-based CI for polar bears. When coupled with demographic and ecological data, lipid content may hold promise for efficiently assessing and monitoring condition of AF polar bears in a rapidly changing Arctic. Monitoring the condition of AFs is critical as it is pregnant and lactating AFs and their cubs that are of greatest concern with respect to climate change impacts (Robbins et al. 2012b). Moreover, because remotely collected samples can be obtained using biopsy darts (Pagano et al. 2014), body condition assessments can be accomplished relatively noninvasively, which may be preferential in more remote areas and for other concerns (Peacock et al. 2011; Vongraven et al. 2012). Though our findings are encouraging, we have also identified limitations of the lipid content CI that warrant further research.

The lipid content CI for capture biopsies is supported by significant relationships to established CIs. Lipid content of AFs varied across FI scores, as did established CIs except for skull width. Lipid content of AFs was also correlated with the published CIs, except skull width, although not as strongly as these CIs were correlated with one another. BMI, BCI, and storage energy, however, are calculated from one or more of the same variables (body mass and/or length). Thus, it is not surprising that they showed stronger correlations with each other than with lipid content. BIA values obtained from SB bears in 2012 were not predictive of lipid content. BIA resistance measures are used as indicators of total body water (TBW) content, which often varies inversely with total fat (TF) content (Gales et al. 1994). However, our finding of no correlation should be interpreted cautiously as (i) the sample size was small, (ii) the relationship between TBW and TF can vary interspecifically (e.g., Reilly and Fedak 1990), and (iii) BIA has yet to be calibrated in the field for use with polar bears. Lipid content differences with FI scores were not as strong for AMs and immature bears; however, for immature bears, this was also the case for all measured CIs. Additionally, the established CIs were not strongly or not significantly correlated with one another for immature bears. These findings may be related to differential use of fat as a stored energy depot between age/sex classes. Specifically, AFs tend to lose/gain more body mass as fat during fasting/hyperphagia than other classes; previous work demonstrated that fasting AFs lost 43% body mass, of which 93% was body fat (Atkinson and Ramsay 1995). In contrast, fasting adult and juvenile males lost 19% body mass, of which only 50% was body fat (Atkinson et al. 1996). Thus, lipid content may be a more suitable CI for AFs than for AMs and immature bears.

If adipose lipid content is reflective of body condition, it should also exhibit certain biological and ecological differences. One prediction, confirmed previously in WH polar bears, is that AFs have higher fall lipid content than AMs due to the timing and higher reproductive costs for AFs (Thiemann et al. 2006). Here, we found a similar although not significant pattern, likely due to low fall sample sizes. Another prediction was that AF lipid content is lower in spring than fall due to reproductive fasting. This was confirmed previously in WH bears (Thiemann et al. 2006) and now also in SB bears. Similarly, we found the greatest decline in lipid from fall to spring for solitary, presumably pregnant, fall AFs (˜16%) compared to AFs with cubs, AMs, and immature bears (10–15%), which is consistent with different fasting lengths among these groups (Stirling et al. 1999).

Lipid content of SB capture biopsies tended to be lower than reported previously for SB and WH polar bear capture biopsies (Thiemann et al. 2006). The previous study used full-depth capture biopsies (down to the muscle), whereas the current study did not. Both studies found higher lipid content in inner versus outer biopsy sections. Thus, the exclusion of a small portion of inner tissue may have slightly biased our lipid content to be lower. Our results may also be related to longer-term storage, and potentially greater desiccation. However, lipid content differences between studies are more likely due to different sampling dates. Previous SB collections were from early April–early May, whereas our data spanned early March–early May. We have shown that the lipid content declines dramatically (˜20%) from early March to May and then subsequently rises again. Thus, small variation in sampling dates may have large impacts on lipid content.

Lipid content of SB remote biopsies was lower than for capture biopsies, possibly because lipid content varied with remote dart type and body position where the dart struck (Pagano et al. 2014). Lower lipid content in these potentially more superficially sampled remote biopsies may also be due to not sampling much of the inner layer of the adipose. Adipose closer to the skin may have more connective tissue resulting in lower lipid content (Ylitalo et al. 2001). However, this is not likely a good explanation for our findings as remote and capture biopsy lengths were similar. Killer whale *Orcinus orca* and beluga whale *Delphinapterus leucas* samples obtained from necropsy (large full-depth tissue sample) showed higher lipid content versus those obtained from biopsy darts of the same individuals at similar depths and body positions (Krahn et al. 2004). The authors concluded that lower biopsy dart readings were from lipid seeping from the blubber during dart removal. Here, as the darts fell into the water or onto land, some lipid may have also been washed away, or the sample may have collected small pebbles/dirt, resulting in low-biased lipid readings. We attempted to remove the pebbles/dirt in the lab, but this may not have been sufficient. Because of lower lipid content readings, remote biopsy results cannot directly be compared to those from capture biopsies or harvested samples. The remote biopsy method requires refinement before it may be informative in determining lipid content. This conclusion is supported by the fact that remote biopsy lipid readings did not significantly vary with FI scores. Nonetheless, remotely assigned FIs may not be as robust as FIs obtained from handled bears.

Both the lack of time trends in SB polar bear lipid content from 2004–2011 and increasing trends in AF EG lipid content from 1984–2010 could be due to some combination of changes in CI and/or effects of storage condition. Controlled studies re-analyzing the same samples after different storage times are required to fully understand the potential influence of storage time. However, the composition of fatty acids (FAs), the main lipid components in polar bear adipose and marine mammal blubber tissues, were reported to be stable in seal blubber re-sampled after up to 6 years at −25°C (Lind et al. 2012), supporting the stability of lipid components in adipose tissues. In agreement, subjective oxidation class did not explain a significant amount of the variation in fatty acid patterns in the 1984–2011 EG dataset (McKinney et al. 2013) Thus, it is unlikely that the lack of SB trends (storage for up to 8 years at −80°C) and the increasing trend in EG females (storage for −20°C for up to 27 years) were driven by substantial confounding effects of long-term storage. We therefore suggest that it is more likely that the trends reflect true changes in CI. Our results suggest that both capture biopsies and harvest samples can be used for studies using lipid content as a CI, which broadens the utility of harvest samples collected in remote subpopulations with poor/expensive research access. Harvest samples can also constitute valuable long-term archives provided storage condition impacts have been fully ruled out, and therefore may be useful in monitoring CI changes over time.

Changes in body condition of polar bears, and ultimately reproductive success and overall survival, are being driven by reduced access to, or abundance of, food resources (Rode et al. 2012). High Arctic Greenland and Canadian Archipelago polar bear habitats are predicted to temporarily improve as multiyear ice-covered areas, with lower primary production, are replaced by annual ice (Stirling and Derocher 1993); concomitantly, a change in ice habitat may alter the prey base. Increasing lipid content in AF EG polar bears between 1984 and 2010 supports this hypothesis, although the influence of storage condition must still be fully evaluated. We recently demonstrated that EG diets shifted over this period from predominately Arctic seals (ringed seals *Pusa hispida*) to sub-Arctic seals (hooded seals *Cystophora cristata* and harp seals *Pagophilus groenlandicus*) (McKinney et al. 2013), supporting a link between increasing body condition and additional food resource availability. Similarly, Davis Strait polar bears have shown higher survival rates and subpopulation size due to an increasing abundance of harp seals (Peacock et al. 2013), although this region at the southern end of the species range has historically been characterized by only annual ice. Declines in SB body size, cub recruitment, and cub, and AF survival have occurred concomitant with sea ice declines in the region from 1982–2006 (Regehr et al. 2010; Rode et al. 2010). Variation in summer/fall sea ice availability, here, was linked to interannual (2004–2011) variation in SB polar bear lipid content. The lack of a temporal decline in SB polar bear lipid content may be due to variance obscuring the trend within this short time series, or to a changing relationship between sea ice and body condition. Similarly, we found no decline in skull width, except for in immature bears, unlike previous declines reported for immature and AF SB bears from 1982 to 2006 (Rode et al. 2010). We are currently investigating the relationship of body condition (lipid content) to diet (fatty acid profiles), habitat use (satellite tracking data) and continued changes in climate and sea ice conditions in the SB subpopulation.

### Recommendations

To enable cross-population and temporal comparisons, researchers should ensure consistency in biopsy collections to reduce lipid content variation, including considerations such as analyzing fat biopsy samples measured to standard length from the skin tab, sampling fully perpendicular to the body surface, and consistent annual collection dates. Controlled studies on tissue portions from the same individual are necessary to completely rule out an influence of storage conditions (time, temperature, container type). Regarding FI, virtually no bears were assigned values of 1 or 5, potentially limiting the ability to elucidate patterns from this index. Researchers and hunters who use the 5-point FI scale should avoid using noninteger values (e.g., 1.5) and instead use all points, if possible. Over the last decade, the view of adipose tissue has changed dramatically from an inert energy reservoir to being recognized as an endocrine organ that produces numerous proteins (adipokines) with broad biological activities. Several factors have now been discovered, particularly leptin, which is related to body fat mass as well as other factors (Hissa et al. 1998; Spady et al. 2009). We therefore recommend that the lipid content CI could be examined for relationships to such biomarkers.

## Data Accessibility

The lipid content data is archived with the Polar Data Catalogue (http://www.polardata.ca).

## Supporting Information

**Appendix S1**. Supplementary methods, results, and discussion, Figures S1 and S2 **Figure S1**. Lipid content extracted from polar bear adipose (top panel) and National Institute of Standards and Technology standard reference material (SRM) 1945 (pilot whale blubber homogenate) (bottom panel) by initial sample mass. **Figure S2**. Adipose lipid content (±SE) extracted from equal length inner (near muscle) and outer (near skin) adipose subsections of capture biopsies from polar bears from the Southern Beaufort (SB) Sea subpopulation from spring 2010. Significant differences in lipid content are indicated by different letters.
