# Supplementary material for: Validation of adipose lipid content as a body condition index for polar bears
Source: Ecol Evol. 2014 Jan 23;4(4):516–27. doi: 10.1002/ece3.956 (PMC3936397; doi:10.1002/ece3.956)
Supplement: Supplementary file 1 — Appendix S1. Supplementary methods, results, and discussion, Figures S1 and S2 Figure S1. Lipid content extracted from polar bear adipose (top panel) and National Institute of Standards and Technology standard reference material (SRM) 1945 (pilot whale blubber homogenate) (bottom panel) by initial sample mass. Figure S2. Adipose lipid content (±SE) extracted from equal length inner (near muscle) and outer (near skin) adipose subsections of capture biopsies from polar bears from the Southern Beaufort (SB) Sea subpopulation from spring 2010. Significant differences in lipid content are indicated by different letters. [file ece30004-0516-sd1.docx]

**Appendix S1: Supplementary methods, results and discussion, Figures S1 and S2**

**Materials and methods**

*Sea ice measures*

Ice-free days (*IFD*) was defined as the number of days each year for which mean sea ice concentration (using 139 25×25 km grid cells) was <50% (Regehr *et al.* 2010) based on passive microwave satellite imagery (National Snow and Ice Data Center, Boulder, CO, USA (ftp://sidads.colorado.edu/pub/). *Melt* was defined as the number of days between the onset of summer melt and fall reformation of ice each year, derived from a modification of the algorithm of Markus, Stroeve & Miller 2009; Julienne Stroeve, personal communication). We developed biologically plausible candidate models, used Akaike’s Information Criterion values (AIC_c_) to determine top models for each sex class and considered models with ΔAIC_c_ values >2.0 to measurably differ in information content (Burnham & Anderson 2002). Year and ice availability metrics were not included in the same model because of their potential to reflect different temporal scales (Rode *et al.* 2012). We assessed potential collinearity in independent variables using measures of tolerance and variance inflation factors (McCullagh & Nelder 1989).

**Results**

*Validation of the laboratory method*

The average lipid content of SRM 1945 was 65.1 ± 8.8% (sd). Performing a third extraction did not recover additional lipid (<1%), indicating that the double extraction was sufficient. Lipid content recoveries for individual samples were checked using the internal standard, 5-α-cholestane. We initially thought 5-α-cholestane might not be fully representative of analytes because it is not derivatized during the transesterification reaction that converts FAs to FAMEs. However, when 13:0, 23:0 and 5-α-cholestane were taken through the procedure, they showed identical recoveries (*n* = 6 replicates). Thus, the derivatization reaction goes to completion, and 5-α-cholestane was indeed a suitable internal standard. Calculated recoveries for the entire procedure averaged 103 ± 10%, indicating that no recovery correction is necessary for lipid content determination. Lipid content values exhibited no relationship with sample weight using subsections of the BB polar bear sample ranging from ~0.005 to 0.2 g (Spearman *r*^2^ = 0.24, *p* = 0.08), nor in aliquots of SRM 1945 homogenate ranging from ~0.005 g to an even larger 0.5 g (*r*^2^ = 0.002, *p* = 0.91) (Fig. S1). Capture biopsies divided into equal length subsections were higher in lipid content for inner (54.76 ± 4.08%) versus outer (33.74 ± 4.20%) adipose tissue (*z* = 4.0, *p* < 0.001) (Fig. S2).

**Discussion**

We found high accuracy of NIST SRM1945 lipid content readings relative to the consensus value of 71.9 ± 1.3% lipid (Kucklick *et al.* 2010). Our values averaged >90% of this published lipid content or ‘total extractable organics’ value. High precision was indicated by a low RSD of 14%, even though the sample sizes were small (~0.05 g). Repeatability was also high as indicated by internal standard recoveries. Lipid content determinations were reliable even for very small biopsies.

**Fig. S1.** Lipid content extracted from polar bear adipose (top panel) and National Institute of Standards and Technology standard reference material (SRM) 1945 (pilot whale blubber homogenate) (bottom panel) by initial sample mass.

**Fig. S2.** Adipose lipid content (± SE) extracted from equal length inner (near muscle) and outer (near skin) adipose subsections of capture biopsies from polar bears from the Southern Beaufort (SB) Sea subpopulation from spring 2010. Significant differences in lipid content are indicated by different letters.
